# Screening for pancreatic cancer in high-risk individuals using MRI: optimization of scan techniques to detect small lesions

**DOI:** 10.1007/s10689-024-00394-z

**Published:** 2024-05-11

**Authors:** Bas Boekestijn, Shirin Feshtali, Hans Vasen, Monique E. van Leerdam, Bert A. Bonsing, J. Sven D. Mieog, Martin N. Wasser

**Affiliations:** 1 Department of Radiology, Leiden University Medical Center, Leiden, The Netherlands; 2 Department of Gastroenterology and Hepatology, Leiden University Medical Center, Leiden, The Netherlands; 3 Department of Surgery, Leiden University Medical Center, Leiden, The Netherlands

**Keywords:** Pancreatic cancer, Screening, High-risk individuals, MRI, Early detection

## Abstract

Pancreatic cancer has a dismal prognosis in the general population. However, early detection and treatment of disease in high-risk individuals can improve survival, as patients with localized disease and especially patients with lesions smaller than 10 mm show greatly improved 5-year survival rates. To achieve early detection through MRI surveillance programs, optimization of imaging is required. Advances in MRI technologies in both hardware and software over the years have enabled reliable detection of pancreatic cancer at a small size and early stage. Standardization of dedicated imaging protocols for the pancreas are still lacking. In this review we discuss state of the art scan techniques, sequences, reduction of artifacts and imaging strategies that enable early detection of lesions. Furthermore, we present the imaging features of small pancreatic cancers from a large cohort of high-risk individuals. Refinement of MRI techniques, increased scan quality and the use of artificial intelligence may further improve early detection and the prognosis of pancreatic cancer in a screening setting.

## Introduction

Pancreatic ductal adenocarcinoma (PDAC) has a dismal prognosis. Over half of patients with PDAC are diagnosed with already metastasized disease and a 5-year survival of 3.2%. However, patients with localized disease (confined to primary site) have a 5-year survival of 44.3% [1]. Thus, early detection can greatly improve survival. Screening for PDAC in the general population is not feasible due to the low incidence of PDAC and the limited accuracy of current screening techniques. However, surveillance programs in populations with hereditary or familial pancreatic cancer have shown increased survival [2–5]. Furthermore, treatment of patients with PDAC detected at stage 1 A leads to greatly improved prognosis, with 5-year survival up to 84% and patients are more often diagnosed with stage 1 A disease, probably related to improved early detection and diagnosis [6]. Patients diagnosed with a PDAC with a size of 10 mm or less have better survival than patients with a PDAC of 11–20 mm [7]. Thus, the goal of surveillance in high-risk individuals (HRIs) is not only to diagnose localized disease before metastases have occurred but also to detect lesions at the smallest possible size or even precursor lesions.

Current guidelines for surveillance in HRIs recommend magnetic resonance imaging (MRI) with magnetic resonance cholangiopancreatography (MRCP) and/or endoscopic ultrasound (EUS) as the primary imaging tools [8, 9]. Unfortunately, these guidelines provide limited guidance on MRI screening protocols. Only recently did the Pancreatic Cancer Early Detection (PRECEDE) consortium publish recommendations for a standardized MRI protocol [10]. The PRECEDE consortium includes members from forty sites in North America, Europe, and Asia; therefore, since all these centers use different sequences and various systems from different vendors, the standardized protocol proposes minimal criteria for necessary sequences. It is based on sequences used for imaging the upper abdomen in general, not on dedicated imaging of the pancreas. Such a standardized protocol is very helpful but may not be sufficient for the detection of small pancreatic cancers in an HRI surveillance program.

In screening for small lesions in the pancreas, pancreas-specific techniques should be used with high resolution, high contrast difference between normal and abnormal pancreatic tissue and with no or limited movement artifacts. Various MRI sequences aid in the detection of pancreatic lesions, but they also detect secondary signs such as stenosis and dilatation of the pancreatic duct.

In this review, we will discuss state-of-the-art MRI techniques and sequences that enable the early detection of malignant pancreatic lesions in HRIs at sub centimeter sizes. Furthermore, we demonstrate the imaging findings of small pancreatic cancers with MRI in our surveillance cohort of HRIs.

### Advances in MRI hardware and software

In the last decade, a plethora of advances in MRI hardware and software have drastically improved image quality and have made MRI a reliable modality for surveillance. Some of the major contributors to the recent development of MRI techniques are described below.

### Magnetic field strength

Increases in magnetic field strength provide a higher signal-to-noise ratio, which can be utilized to scan with higher spatial resolution or with faster acquisition times. This has driven the development of MRI scanners with greater magnetic field strength. However, increased magnetic field strength also exacerbates artifacts encountered during MRI, such as magnetic field inhomogeneity, susceptibility, and chemical shift artifacts. Most clinically available scanners have a field strength of 1.5 or 3 Tesla (T). Studies have shown an improved signal-to-noise ratio and spatial resolution using 3T machines for both imaging of the pancreas and MRCP, without a significant increase in artifacts [11, 12].

The use of high-field MRI is attractive for the detection of small PDAC lesions because of the potential further increase in spatial resolution. Imaging at 7 Tesla has successfully been implemented in various applications, such as musculoskeletal and neuroradiology. The abdomen, however, provides additional challenges due to increased magnetic field inhomogeneities and motion artifacts related to respiratory and gastrointestinal movement as well as vascular pulsation. Especially in the region of the pancreas, field inhomogeneity and motion can be severe. To date, no clinical research dedicated to 7 Tesla MRI of the pancreas has been published. Comparison between 1.5T, 3T and 7T on general imaging of the abdomen shows detrimental artifacts on 7T, outweighing the benefit of increased spatial resolution [13]. Therefore, MRI at 3 Tesla currently appears to be the optimum for imaging the pancreas.

### Reducing motion artifacts

Imaging of the abdomen is very susceptible to motion due to multiple sources, of which the respiratory cycle is one of the major culprits. One of the most effective methods to reduce motion is simple instruction of the patient and scanning during breath holds. The use of a belt or bellows placed on the body of the patient or camera systems to detect respiration enables scanning with respiratory triggering or gating, allowing more accurate timing of data acquisition. Various efficient scan techniques, referred to as ultrafast imaging, have been developed that significantly reduce scan times and thus help reduce motion artifacts [14]. MRI gathers data in the so-called k-space, i.e., the temporary matrix in which data from digitized MR signals are stored during data acquisition; each point in the k-space contains spatial frequency and phase information about every pixel in the final image. The manner in which data from the k-space is processed to obtain images is important. Most sequences read the k-space in a Cartesian (rectilinear) grid. However, some techniques use a radial pattern through which oversampling of the k-space center allows for motion artifacts to be reduced. Techniques such as these even allow sequences to be acquired while the patient is freely breathing, which is especially useful in patients unable to suspend respiration [15].

### Acceleration of image acquisition

Reduction of image acquisition times while maintaining high spatial resolution results in reduction of motion artifacts and increase in diagnostic confidence.

Compressed sensing is a technique that has become clinically available only in recent years. This technique accelerates MRI acquisition by acquiring less data through undersampling of the k-space and algorithms developed with the aid of artificial intelligence (AI) are used to reconstruct the image. This technique is applicable to many MRI sequences and is capable of reaching up to tenfold acceleration rates of image acquisition [16]. For abdominal imaging, compressed sensing has also been shown to accelerate acquisition without impairing image quality [17]. Furthermore, instead of only using the technique as a method of reducing scan time, it can also be used to acquire more spatial resolution in the same or even a reduced scan time [18, 19]. Due to the combination of increased spatial resolution, reduced scan times and therefore potentially reduced motion artifacts, compressed sensing provides a large leap in improving MRI of the pancreas.

Other AI applications, are now becoming integrated into the MRI imaging chain [20, 21]. They can be applied during image generation, reconstruction and postprocessing of the image data, which may result in a significant reduction in motion sensitivity and higher resolution [22].

With AI reconstruction technology, another application will become possible in the near future: synthetic image generation. Synthetic MRI is based on parametric maps. In this application, the MR scanner is not used to acquire images but to measure magnetic properties in the tissues. By synthesizing a quantitative map of tissue properties from MR signal evolution over the signal acquisition trajectory, different images may be generated from these tissue properties [23].

### MRI sequences

This section describes the various sequences that contribute to multiparametric MRI of the pancreas.

### T1-weighted sequences

As one of the corner stones of MRI, T1-weighted sequences play a major role in imaging of the pancreas. T1-weighted images not only provide anatomical information and potential for lesion detection but also enable the characterization of lesions due to varying T1 relaxation times and thus varying signal intensities of various substances. For the same reason, T1 lends itself to imaging with gadolinium-based contrast media due to its short T1-relaxation time, providing a hyperintense signal on T1-weighted sequences. Images are usually acquired with a gradient recalled echo (GRE) technique, which, because of low echo times and low flip angles as well as low repetition times, provides rapid signal acquisition. This makes it possible to quickly image and record multiple phases after administration of gadolinium contrast. A shortcoming of the GRE technique is its susceptibility to inhomogeneities of the magnetic field, leading to artifacts.

T1-weighted GRE sequences can be acquired in a two-point manner with two echo times in which protons in water and fat are imaged in the same or opposed phase. This enables estimation of fat content based on the amount of signal drop-off on the opposed phase. Focal fatty replacement of the pancreas has been associated with various conditions, such as obesity, diabetes mellitus, chronic pancreatitis, hereditary pancreatitis, or obstruction of the pancreatic duct by calculus or tumor [24]. Fatty infiltration may involve interlobular fat, i.e., the presence of fat-containing cells between pancreatic lobules, lipid droplets within pancreatic acinar cells or islets, pancreatic acinar-to-adipocyte trans differentiation, and replacement of dead pancreatic acinar cells [25]. Intrapancreatic fat is often labile, and fatty pancreas disease is, in principle, reversible. A progressive increase in intrapancreatic fat, however, can be a herald of pancreatic cancer. Focal fatty infiltration may be associated with pancreatic cancer, either as a causative factor [26] or as a secondary sign due to focal inflammation, parenchymal atrophy and fat replacement around tumors.

**Fig. 1:**
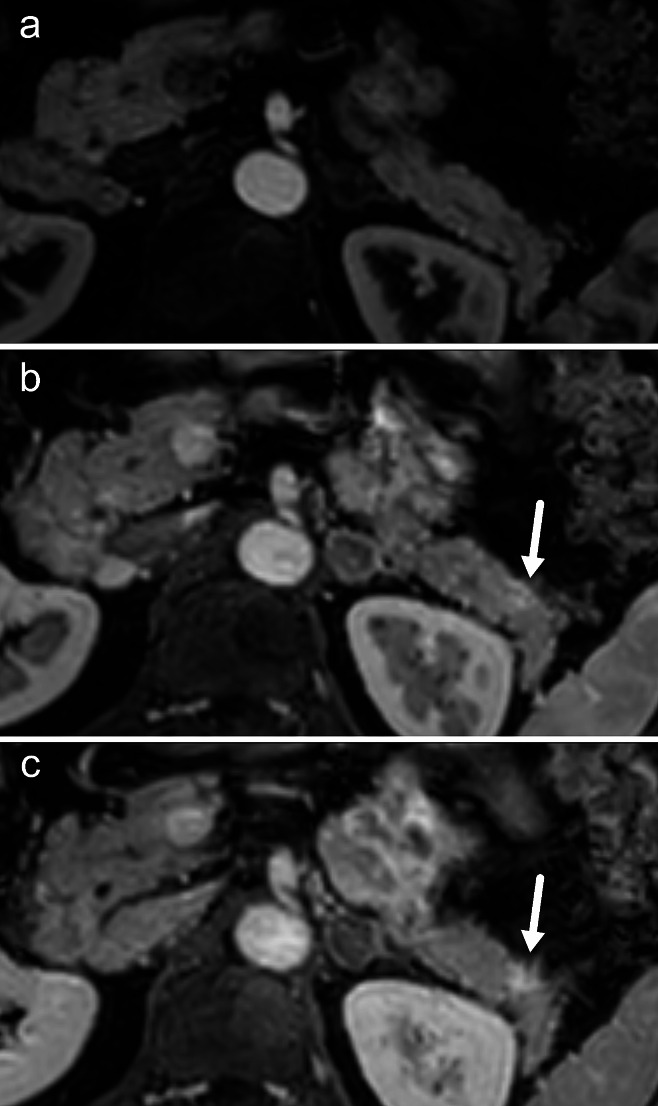
PDAC of 8 mm in the pancreatic tail on fat suppressed T1 after contrast. The tumor is isointense with normal pancreatic parenchyma in the arterial phase (a) but shows progressive enhancement in later phases (b and c)

Decomposition of water and fat signals through multipoint MRI can also be used as a fat suppression method, as first described by Dixon [27]. Various modifications of this technique have proved to be robust methods for fat suppression not only in T1-weighted GRE imaging but also in other sequences [28]. T1-weighted sequences with fat suppression are the ideal platform for imaging after the administration of a gadolinium-based contrast medium. The normal pancreatic parenchyma shows the most enhancement in the late arterial phase, after which the amount of enhancement decreases over the portal venous and late phases. PDAC, on the other hand, usually shows little enhancement in the late arterial phase and increased contrast retention in the late phase (Fig. 1). This enhancement pattern is due to the relatively high amount of fibrosis present in PDAC, increasing the extracellular space, which retains contrast medium in late imaging phases. However, these characteristics are variable depending on the amount of angiogenesis and fibrosis in the tumor [29].

**Fig. 2:**
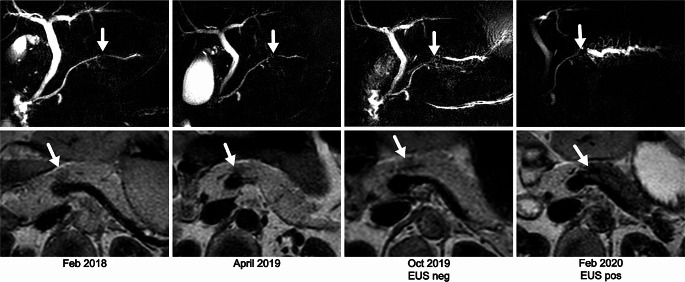
Focal area of decreased signal intensity in the pancreas neck on T1 turbo field echo with presaturation pulse already present in Feb 2018, April 2019, and October 2019 (lower panel), probably indicating focal inflammation, while on EUS, no abnormalities were seen. On MRCP, the PD showed irregularities and subtle dilatation of the proximal duct (upper panel). Finally, in February 2020 (2 years after the start of the parenchymal changes), obvious dilatation of the proximal PD and side branches was visible, combined with diffuse decreased T1 signal intensity of the pancreatic parenchyma in body and tail due to duct obstruction. By that time, a 7 mm tumor was found on EUS at the site of obstruction of the pancreatic duct

The normal pancreatic parenchyma has a high signal intensity on unenhanced T1-weighted images due to a large amount of aqueous protein in the acini as well as abundant endoplasmic reticulum and various paramagnetic ions in the protein-producing acinar cells [30]. Cystic and solid lesions as well as abnormal pancreatic parenchyma due to fibrosis or inflammation have a lower signal intensity on T1. In our surveillance program for high-risk individuals, we utilize this phenomenon through an extra T1-weighted sequence with a presaturation pulse, a T1 turbo field echo sequence (a sequence similar to magnetization prepared rapid gradient echo techniques). By optimal timing of the pulse, contrast between normal pancreatic tissue and lesions can be increased. In a previous analysis, we found this sequence to be able to sometimes detect abnormalities of a PDAC lesion before it was visible on other sequences or EUS [31] (see Fig. 2*)*.

### T2-weighted sequences

Another cornerstone of MRI. T2 provides great anatomical detail, delineation of the pancreatic duct and visualization of cystic lesions. Multishot turbo/fast spin echo and single shot fast spin echo are the most common techniques used for T2-weighted imaging in the abdomen. There is a lack of research dedicated to the image quality of pancreatic imaging. However, research suggests that multishot sequences can yield better image quality than single shot sequences in MRI of the liver, while single shot sequences deliver better performance in detecting solid liver lesions [32]. Radial k-space acquisition sequences are another option. In our own HRI cohort, we found that such a sequence provides better image quality with fewer artifacts compared with multishot sequences in MRI of the pancreas. Research on MRI of the liver has shown both increased image quality and lesion detection of radial k-space acquisition compared to conventional T2-weighted sequences [33].

**Fig. 3:**
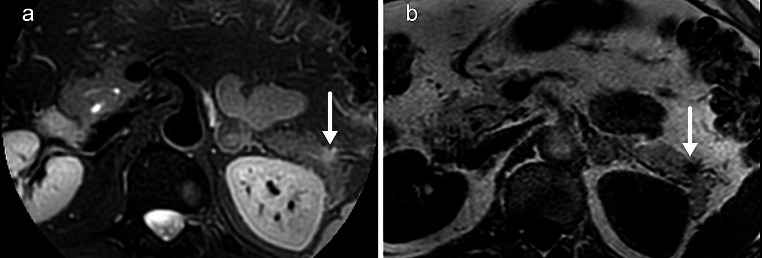
Small hyperintense lesion in the pancreatic tail on fat-suppressed T2-weighted turbo-spin echo sequence with radial k-space acquisition (a). B shows the lesion on T1 with presaturation pulse for comparison

Fat-suppressed T2-weighted images are very helpful in dedicated imaging of the pancreas. Cysts, inflammation, sometimes induced by a small PDAC, and solid lesions often demonstrate increased signal intensity on T2 (Fig. 3). Fat suppression increases delineation of such lesions compared to normal T2-weighted images. Two different methods of fat suppression are often used in abdominal imaging. The chemical shift method relies on the different resonance frequencies of protons in water and fat, using a spectrally selective radiofrequency pulse to null the signal of fat. This method is susceptible to heterogeneity in the magnetic field, leading to incomplete suppression, which often occurs in the region of the pancreas. Inversion recovery sequences are less susceptible to magnetic field heterogeneity and can provide homogeneous suppression even in the region of the pancreas but at the cost of lower spatial resolution [32]. There are also hybrid techniques that combine selective radiofrequency and inversion recovery pulses. In our own pancreas surveillance protocol, we have had the most success with such a sequence in the form of spectral presaturation with inversion recovery on a radial k-space sampled T2-weighted sequence.

### MRCP

**Fig. 4:**
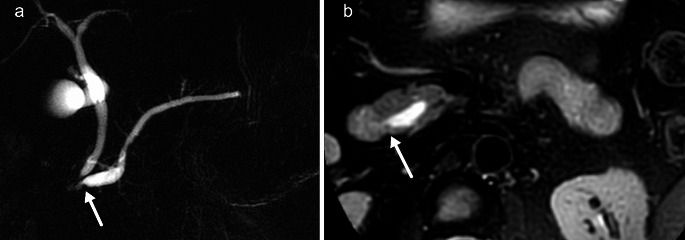
Small tumor partially obstructing the distal pancreatic duct as visible on MRCP (**A**). (**B**) Axial fat-suppressed radial T2-TSE findings

As the best sequence to image the pancreatic duct, MRCP is a necessity in a screening or surveillance protocol for early pancreatic cancer. Studies show that pancreatic duct stenosis and dilatation are often present in early pancreatic cancer [34]. MRCP is acquired with heavily T2-weighted sequences using either a thick slab 2D with multiple coronal slabs (Fig. 4) or a 3D technique. Often, both techniques are combined in MRCP protocols. 3D techniques allow for precise anatomical evaluation of the pancreatic duct and the biliary tree, which, for example, is useful in staging perihilar cholangiocarcinoma. However, in a surveillance protocol for PDAC, the objective is to detect pancreatic duct stenosis and dilation, for which we believe a 2D thick slab sequence is sufficient, reducing scan time.

Secretin is a hormone that induces exocrine secretion of the pancreas, temporally increasing the diameter of the pancreatic duct. Secretin-enhanced MRCP is therefore useful in evaluating complex anatomical variations of the pancreatic ductal system and sphincter of Oddi dysfunction and can improve the diagnosis of early signs of chronic pancreatitis [35]. The added benefit to visualizing duct stenosis and dilatation is only minor, but imaging time and costs are increased. Consequently, secretin-enhanced MRCP is not recommended in a surveillance setting for PDAC.

### Diffusion-weighted imaging

Diffusion weighted imaging (DWI) utilizes the random motion of water molecules to analyze the biology of tissues. One of the major hindrances to the motion of water molecules is the cell membrane, and therefore, highly cellular tissues produce diffusion restriction. DWI sequences use T2-weighted images with fat suppression and apply multiple magnetic field gradients with a certain b-value representing the strength and timing of the gradient. At higher b-values, the signal remains mainly in tissues with restricted diffusion of water molecules. Apparent diffusion coefficient (ADC) values are calculated based on the slope of signal decay over the different b-values to quantify diffusion, in which lower values represent increased diffusion restriction.

In the abdomen, echo planar imaging is the technique most often used for DWI, and both breath-hold and free-breathing techniques are possible. There is no consensus on the b-values to be used for imaging the pancreas. However, it is recommended to include at least a b-value over 100 s/mm^2^ to null perfusion effects and a high b-value acquisition up to 1000 s/mm^2,^ as higher values lead to a decreased signal-to-noise ratio [36]. In our protocol, we opted to use b-values of 0, 100 and 800 s/mm^2^, as we found b-values of 1000 s/mm^2^ to exacerbate artifacts and noise compared to 800 s/mm^2^.

**Fig. 5:**
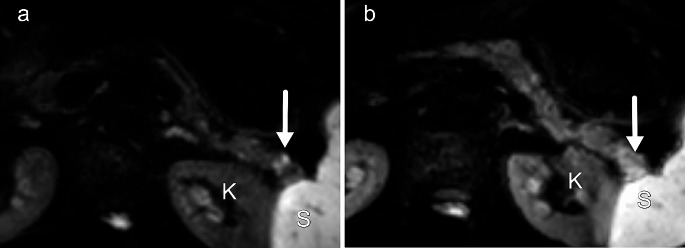
Diffusion-weighted image (b 800) of a small PDAC in the pancreatic tail. In July (**a**), a small focus of high signal was visible, but EUS was negative. Six months later (**b**) a larger area of high signal was visible. On pathology, the actual size of the tumor was only 6 mm, and the larger aspect of the tumor on DWI was due to inflammation and fibrosis around the tumor K = left kidney, S = spleen

PDAC usually presents with diffusion restriction and low ADC values because of high cellularity and fibrosis in the tumor (Fig. 5). Therefore, DWI helps with the detection and characterization of lesions. However, both diffuse and focal pancreatitis also demonstrate varying degrees of diffusion restriction due to inflammation and fibrosis. Various studies have compared the ADC values of PDAC and focal mass-forming pancreatitis and have reported conflicting results [36]. For this reason, DWI cannot be used to differentiate between these two entities.

#### MRI protocol for imaging the pancreas in surveillance

An MRI surveillance protocol for pancreatic cancer should carefully be constructed to provide optimum image quality and include the proper sequences to detect small lesions, without false positive findings. Table 1 summarizes the minimal sequences and slice thicknesses recommended by the PRECEDE consortium. Specific recommended sequences, repetition and echo times are omitted because these will differ based on vendor and various other parameters. This table also shows the protocol as used in our surveillance program of HRIs.

**Table 1:** MRI and MRCP minimal sequences for pancreatic cancer surveillance as recommended by the PRECEDE consortium (8) and protocol used in the Leiden University Medical Center

PRECEDE protocol recommendations: sequence, imaging plane	Slice thickness (mm)	Protocol at Leiden University Medical Center: sequence, imaging plane	Slice thickness (mm)
T2-weighted		T2-weighted	
Axial	4	Axial oblique radial k-space acquisition	3
Coronal	4	Axial oblique radial k-space acquisition with SPIR FS	3
		Coronal T2 TSE	3
MRCP		MRCP	
2D slab coronal	40	Radial coronal thick slab	40
3D coronal	1		
T1-weighted		T1-weighted	
		Axial oblique TFE with presaturation pulse	3.5
		Axial oblique DIXON 3D GRE	
2D In and opposed phase axial	4	In and opposed phase	3
3D FS Axial precontrast	3	Precontrast FS	3
Axial postcontrast (arterial, venous, and late venous phase)	3	Postcontrast FS (arterial, venous and late venous phase)	3
Coronal postcontrast venous late phase	3		
DWI/ADC		DWI/ADC	
Axial with b-values of 50, 100 and 800 s/mm^2^	5	Axial oblique with b-values of 0, 100 and 800 s/mm^2^	5

**Fig. 6:**
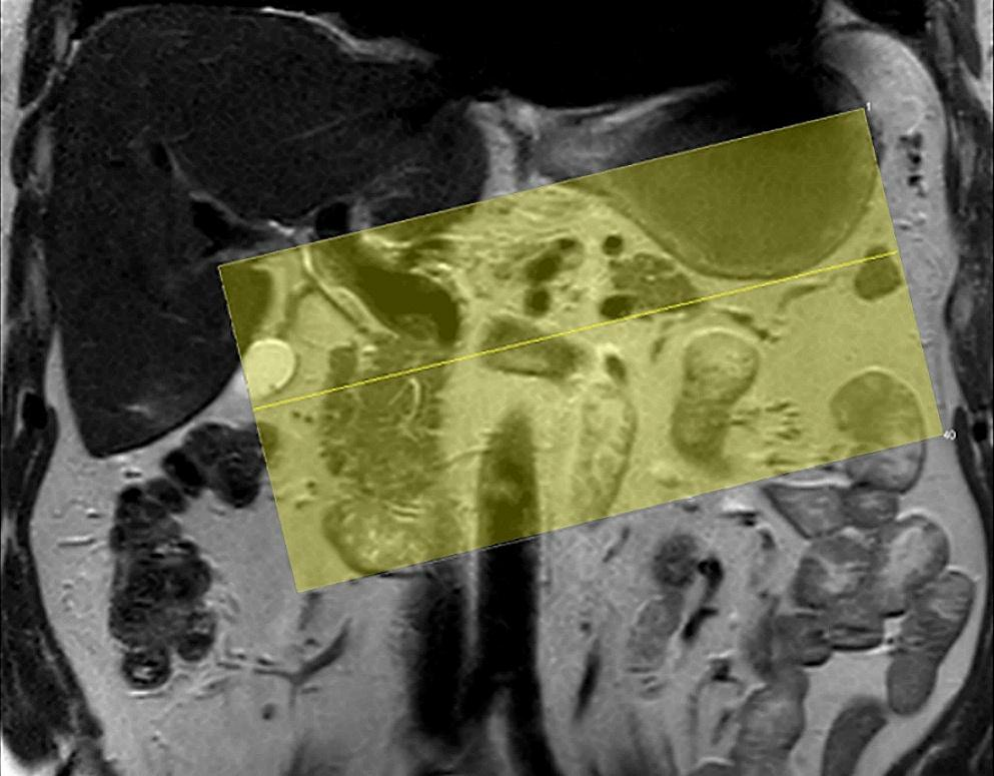
Axial oblique scan plane, small field of view of 220 × 220 mm

The image quality of the pancreatic region can be drastically improved on MRI by scanning with a smaller field of view, greatly increasing spatial resolution. This is routinely performed in MRI of other organs, such as the prostate, but seldom if ever utilized for the pancreas. For the axial sequences in our protocol, we use a small field of view with a size of 220 × 220 mm. Furthermore, we used a slightly oblique plane perpendicular to the course of the pancreatic body and tail, which in most people courses superior toward the splenic hilum (Fig. 6). The oblique axial plane has two benefits. First, it improves the depiction of the pancreatic body and tail, and second, it reduces the number of slices required to image the pancreas. The increased spatial resolution that can be achieved in this manner enables the detection of smaller lesions compared to a larger field of view, which is mostly used in imaging the upper abdomen. To further increase spatial resolution, slice thickness should be considered and reduced as much as possible.

In our institution, we use a 3 Tesla system (Philips Ingenia; Philips Medical Systems, Best, The Netherlands) using a 32-channel torso coil. Axial T1-, T2- and DWI-weighted images acquired on an oblique axial plane with a small field of view of 220 mm. T1-weighted sequences consist of axial multiphase 3D GRE with fat supression through the DIXON method before and after administration (at 20 s, 1 min and 3 min) of a gadolinium-based contrast agent (Dotarem, Guerbet, France), in and opposed phase images and an unenhanced turbo field echo with a 180-degree presaturation pulse with an inversion time of 1200 ms. T2-weighted sequences consist of axial radial k-space sampled images (MultiVane; Philips) with and without fat suppression through spectral presaturation with inversion recovery (SPIR). Compared to a conventional k-space sampling used prior, we found this method to greatly reduce movement between slices. Furthermore, a coronal T2-weighted turbo spin echo sequence of the upper abdomen is included. Axial DWI is acquired with b-values of 0, 100 and 800 s/mm^2^. Last, only thick-slab coronal MRCP is acquired, as this provides ample visualization of the pancreatic duct to evaluate irregularities, stenosis, and dilatation. We omit 3D MRCP sequences because they take a large amount of scan time with only limited additional value. The MRCP- and dynamic contrast-enhanced sequences are performed during a breath-hold of 12 s, and the other sequences are performed using respiratory triggering.

### The Leiden experience with dedicated MRI in surveillance for PDAC in hereditary pancreatic cancer

In 2000, we started a surveillance program in a large cohort of CDKN2A *p16-Leiden* mutation carriers of, at the moment of writing, 347 individuals. Surveillance was initially offered from the age of 45 or 10 years before the youngest age of familial onset, but from 2020 the age of enrolment was lowered to 40 years based on the International Cancer of the Pancreas Screening Consortium (CAPS) guideline [8]. The mean age at start of surveillance was 48.6 years (range 44.5–55.7). For this annual surveillance, we used a dedicated MRI protocol for imaging of the pancreas. Over time, technological advances have improved the ability of MRI to detect PDAC in HRIs at an early stage. In the last few years, such advances and increased expertise have enabled us to regularly detect PDAC at sub centimeter size.

The results of our surveillance program are summarized in Table 2, which shows the results of screening in the period between 2000 and 2020, as published previously [4, 37]. All PDAC were confirmed by histological analysis. This table also shows the results of screening between 2020 and 2023, a period in which new techniques were implemented for T2-weighted imaging and the MRI protocol was further optimized. In the first period, 36 PDAC tumors were detected of which 30 were detected during regular surveillance imaging and six as an interval tumor. Seven PDAC were T1 stage tumors (< 2 cm) and two were smaller than 1 cm. Between 2020 and 2023, we detected eight cancers, all during regular surveillance imaging: seven were T1 tumors, and four tumors were smaller than 1 cm. All T1 stage tumors were resectable. One of the patients unfortunately was found to have metastatic pancreatic head carcinoma on MRI during the regular yearly surveillance interval. Even in retrospect, we could not detect any abnormalities in the pancreas on the previous examination.

**Table 2:** Results of screening for PDAC in CDKN2A*p16*-*Leiden* mutation carriers

Period	Detected PDAC	Tumors < 2 cm	Tumors < 1 cm	Resectability	5-year survival
2000–2020	36	7	2	83.3%	44.1%
2020–2023	8	7	4	87.5%	N.A.

Since 2000, nine patients underwent surgery for a suspicious lesion that proved not to be PDAC. One patient had an ampullary carcinoma, five had a precursor lesion with low grade dysplasia, one had a neuroendocrine tumor, one had ductal proliferation with chronic inflammation and one patient was found to have diffuse islet cell hyperplasia. Furthermore, multiple HRI’s were found to have focal areas of signal abnormalities on MRI, in many of whom a lesion was also demonstrated on EUS. However, biopsy and/or subsequent follow up did not reveal malignancy. These abnormalities regressed or remained stable over time. If a biopsy was successful, it most often showed chronic inflammation. In a future analysis, we hope to further evaluate such abnormalities, when more follow up is available.

### Imaging characteristics of small (≤ 2 cm) pancreatic cancers on MRI

Although adenocarcinoma in situ or high-grade pancreatic intraepithelial neoplasia (PanIn) lesions can sporadically be visualized with endoscopic ultrasound [38], these lesions are often not visible on MRI. Sometimes indirect signs such as focal pancreatic atrophy [39] and irregularities or stenosis of the pancreatic duct and/or dilated side branches [40] can be seen, indicating the presence of a lesion, even if the tumor itself is not visible. On endoscopic ultrasound, a hypoechoic rim around the tumor may be seen, indicating inflammation on histology [41]. This focal pancreatitis may lead to fibrosis and focal atrophy, which can be seen on MRI. Focal fatty infiltration may also occur, but research on the ability of MRI to reliably detect this is still lacking. MRI is also an excellent tool for the detection and follow-up of pancreatic cysts and demonstrating worrisome features and high-risk stigmata [42, 43]. Additionally, a focal stricture or abrupt cutoff in the pancreatic duct can be visualized with MRI as an early secondary sign of an underlying obstructive tumor [34].

The tumor itself usually presents as a T1-hypointense and slightly T2-hyperintense lesion with diffusion restriction. After contrast administration, the normal pancreatic tissue enhances vividly in the late arterial phase, while PDAC is usually hypointense in this and the portal-venous phase. Depending on the amount of fibrosis in a lesion, enhancement of a PDAC can be increased in the delayed phase. Larger tumors especially show indistinct margins. The fibrosis induced by the tumor often leads to strictures of the pancreatic duct and side branches with upstream duct dilatation. A multicenter study of early pancreatic cancer in Japan found that dilatation of the pancreatic duct was a reliable sign of early pancreatic cancer on MRI, in up to 85.2% of patients with stage 1 disease [38]. Other bodies of research have also demonstrated that PanIn lesions can develop periductal fibrosis and induce strictures of the pancreatic duct [44]. In our own cohort, we previously found similar results, where dilatation of the pancreatic duct was sometimes the only sign of a developing tumor [45]. Considering this, irregularities, strictures, or dilatation of the pancreatic duct in HRIs should be viewed with a high level of suspicion for PDAC, especially in the absence of other causes, such as chronic pancreatitis.

Inflammation and fibrosis surrounding PDAC can be visible on MRI as a T1-hypointense and T2-hyperintense region, occasionally with delayed enhancement due to the increased extracellular space. Although inflammation and fibrosis are frequently scattered throughout a lesion and not separately visible, in some instances, a distinct margin can be visible around a lesion. Areas of focal pancreatitis can also be encountered at imaging in a screening setting, sometimes lacking other suspicious features such as duct abnormalities or an enhancing mass. The T1 turbo field echo sequence with presaturation pulse we utilize seems to be most sensitive for this phenomenon, as focal signal changes were sometimes visible more than a year prior to the diagnosis of small PDAC [31]. The workup of small areas of signal changes on MRI should include EUS with biopsy if possible. If no malignancy is demonstrated, MRI follow-up with a shortened interval is warranted, as an area of inflammation or fibrosis can be induced by a precursor lesion or developing malignancy. When an area of signal changes remains stable or returns to normal, regular yearly surveillance can be continued.

Summarizing these imaging features, five basic abnormalities can be found on multiparametric MRI in early pancreatic cancers:

- Low signal intensity on T1 TFE (magnetization prepared gradient echo).
- High signal intensity on T2 (with FS).
- Diffusion restriction on DWI.
- Delayed contrast enhancement.
- Pancreatic duct abnormalities on MRCP.

**Fig. 7:**
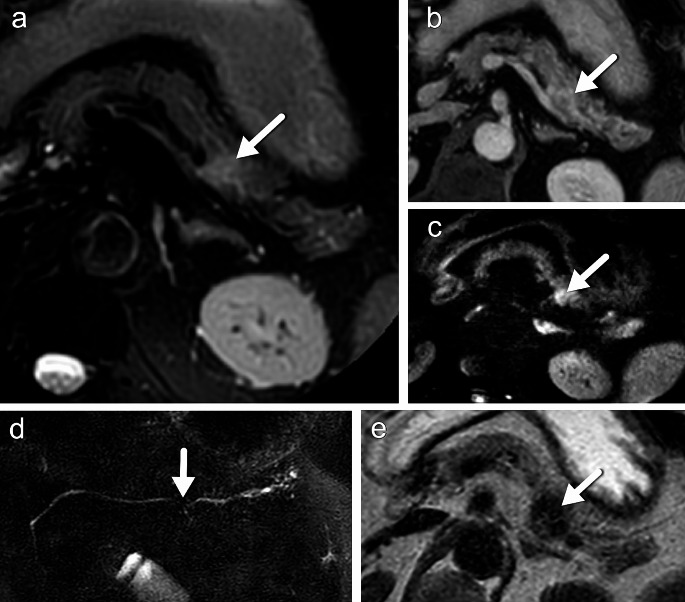
12 mm PDAC in the pancreatic tail on fat suppressed T2 (**A**), late enhancement on T1 after contrast (**B**), DWI (b800) (**C**), MRCP (**D**), T1 turbo field echo (**E**)

**Fig. 8:**
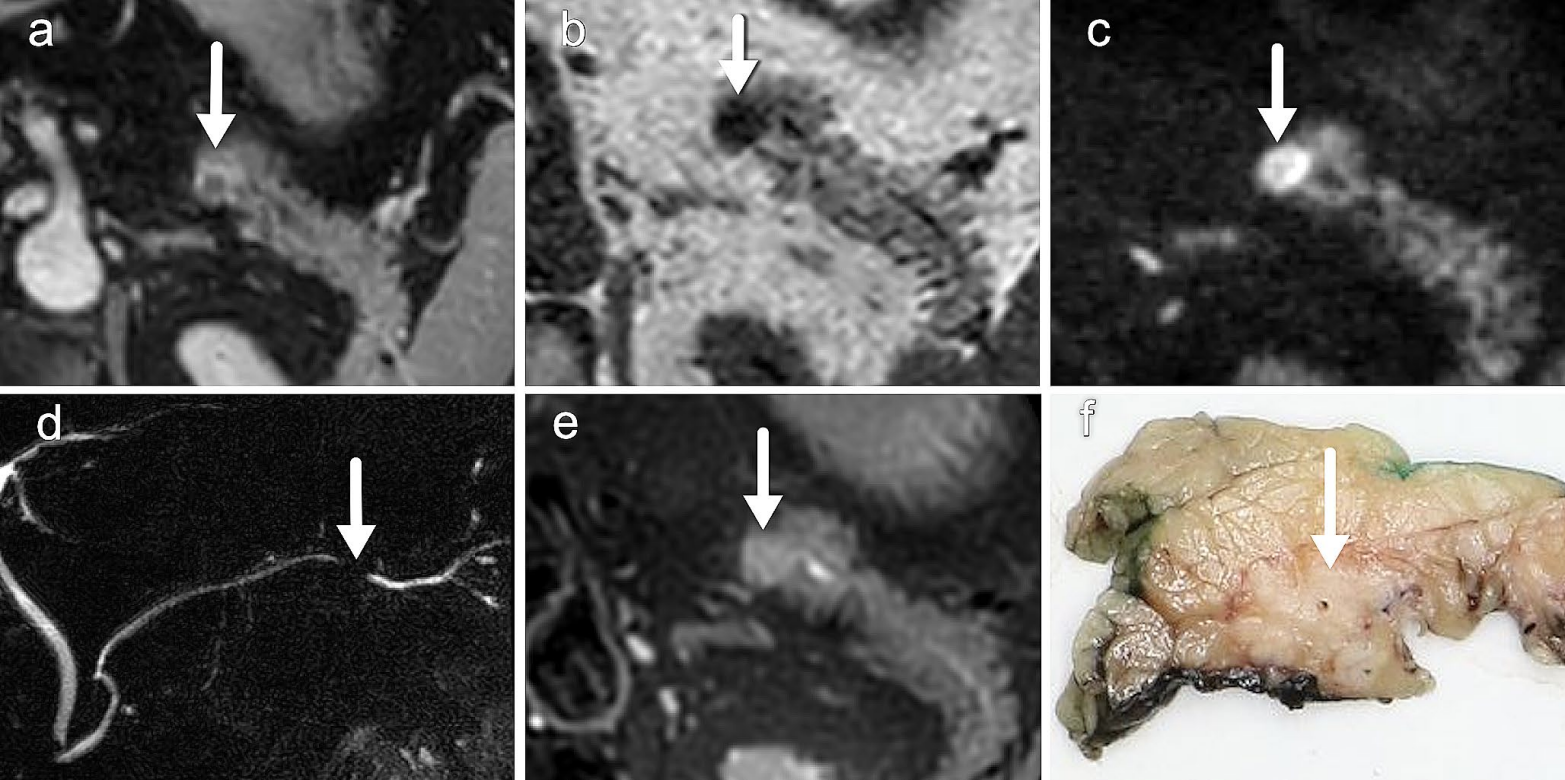
PDAC in the tail as seen on (**a**) late enhancement at T1 after contrast, (**b**) T1 turbo field echo, (**c**) DWI (b800), (**d**) MRCP, (**e**) fat suppressed T2 and (**f**) macroscopic pathology specimen

The more abnormalities are seen on the various sequences, the higher the probability of a malignant lesion. The presence of an abnormality on at least two different sequences also proves that is a true abnormality and is not caused by an artifact. With this set of sequences and minimization of movement artifacts, it is possible to detect small pancreatic lesions (Figs. 7 and 8).

At the moment it is unclear whether there is a significant difference in biological behavior of PDAC between various groups of HRI’s. Development of PDAC from precursor lesions is faster in HRI’s as a whole compared to sporadic pancreatic cancer, but there is not enough data for subgroup analysis [46]. Nonetheless, imaging characteristics of early PDAC and precursor lesions are expected to be similar in the various HRI groups and sporadic pancreatic cancer. Perhaps the lifetime risk of an HRI group correlates to the biological behavior: faster progression to malignancy in specific pathogenic gene mutation carriers with a higher lifetime risk like mutation carriers of the CDKN2A *p16-Leiden* pathogenic variant. Therefore, indeterminate lesions should be handled with greater caution in case of a high lifetime risk individual, as the detection window for early pancreatic cancer may be narrower.

### MRI versus EUS: long-term yield of pancreatic cancer surveillance in high-risk individuals

MRI and EUS are regarded as complementary imaging modalities in screening for pancreatic cancer in HRIs. It is often said that MRI is superior in the detection of cystic lesions and EUS is superior in detecting solid lesions. In a multicenter comparative analysis of EUS and MRI for screening of pancreatic cancer in high-risk individuals [47], MRI, contrary to EUS, was found to be very sensitive for the detection of cystic lesions of any size but could not demonstrate solid lesions. However, in this study, only two solid lesions and nine cysts were detected in 139 HRIs, in which the solid lesions were detected only with EUS. These results were not reflected in our own cohort [4, 37], as we found MRI to be highly accurate in the detection of solid lesions. Aside from the very low numbers of solid lesions detected in this study, a suboptimal MRI protocol can further explain the discrepancy between MRI and EUS. In our view, a dedicated MRI protocol with properly selected sequences, a small FOV and limited artifacts is key to a successful surveillance program and allows for the detection of lesions at the smallest possible size.

### Where should surveillance be performed?

Dedicated screening of HRIs with MRI demands experience and expertise of radiologists. Although no research on reader experience in MRI surveillance of the pancreas in HRIs is available, part of the improvement of screening programs such as our own is attributable to increased reader experience. As radiologists become more familiar with the imaging features of early pancreatic cancer on MRI, they will be better able to detect these features. One of our previous analyses showed that multiple HRIs had direct or indirect signs of PDAC on MRI scans prior to the examination of diagnosis [31]. In recent years, as lesion size at detection decreased, even retrospectively, there were usually no signs of disease on the previous examination.

Another consideration is the value of a multidisciplinary meeting of radiologists, gastroenterologists and surgeons to discuss findings in HRIs. In our institution, we organize a monthly meeting that provides the opportunity for a second reading of dubious findings on MRI and the ability to refine the management of HRIs.

For these reasons, we recommend that whenever possible surveillance should be performed by specialized centers with a team of radiologist, gastroenterologist and surgeons that are experienced in the surveillance of HRI’s. MRI should be performed on a 3T system with a dedicated imaging protocol. When these high-end facilities are not available, surveillance should still be pursued as suboptimal screening is better than none.

### Development of artificial intelligence aided detection

AI shows great promise across the entire field of radiology to aid radiologists in reading examinations and decision making. Also, the possibilities of AI-aided detection of pancreatic cancer in HRIs should be explored. To develop AI, large datasets are necessary to train algorithms. Most cases of symptomatic pancreatic cancer are imaged with computed tomography (CT), and therefore, a large amount of CT data is available. Unsurprisingly, research on AI in pancreatic cancer is most abundant for CT, in which multiple facets of imaging, such as detection, segmentation and malignancy prediction of pancreatic lesions, are being studied [48]. Research on AI for MRI of the pancreas is sparse. Some researchers have investigated methods for the segmentation of pancreatic cancer on MRI [22, 49]. As of yet, no publications of AI-aided detection on MRI are available even for PDAC in the general population. Considering the small lesion size, we aim to detect in HRIs and the relatively small numbers of PDAC lesions that are detected through screening, much work remains to develop clinically available AI-aided detection of PDAC. Nonetheless, due to the promise of AI, this is an endeavor that should be pursued.

## Conclusion

Gradual advancement of MRI techniques has enabled early detection of PDAC in screening settings of HRIs. To achieve detection of lesions at the smallest possible size, a dedicated MRI protocol with optimized sequences, spatial resolution and field-of-view is necessary. Further development of MRI techniques, reader experience and AI can further improve the early detection of lesions and survival of HRIs with pancreatic cancer.

## Data Availability

No datasets were generated or analysed during the current study.
